# Modification of plant cell wall structure accompanied by enhancement of saccharification efficiency using a chemical, lasalocid sodium

**DOI:** 10.1038/srep34602

**Published:** 2016-10-03

**Authors:** Emiko Okubo-Kurihara, Misato Ohtani, Yukio Kurihara, Koichi Kakegawa, Megumi Kobayashi, Noriko Nagata, Takanori Komatsu, Jun Kikuchi, Sean Cutler, Taku Demura, Minami Matsui

**Affiliations:** 1 RIKEN Center for Sustainable Resource Science, Yokohama, Kanagawa 230-0045, Japan; 2 Graduate School of Biological Sciences, Nara Institute of Science and Technology, Ikoma, Nara 630-0192, Japan; 3 Forestry and Forest Products Research Institute, Tsukuba, Ibaraki 305–8687, Japan; 4 Faculty of Science, Japan Woman’s University, Bunkyo-ku, Tokyo 112-8681, Japan; 5 Graduate School of Medical Life Science, Yokohama City University, Yokohama, Kanagawa 230-0045, Japan; 6 Department of Botany and Plant Sciences, Center for Plant Cell Biology, University of California Riverside, 5451 Boyce Hall, Riverside, CA 92521, USA

## Abstract

The cell wall is one major determinant of plant cell morphology, and is an attractive bioresource. Here, we report a novel strategy to modify plant cell wall property by small molecules. Lasalocid sodium (LS) was isolated by chemical screening to identify molecules that affect the cell morphology of tobacco BY-2 cells. LS treatment led to an increase in cell wall thickness, whilst the quantity and sugar composition of the cell wall remained unchanged in BY-2 cells. The chemical also disordered the cellular arrangement of hypocotyls of *Arabidopsis* plants, resulting in a decrease in hypocotyl length. LS treatment enhanced enzymatic saccharification efficiency in both BY-2 cells and *Arabidopsis* plants. Microarray analysis on *Arabidopsis* showed that exposure to LS upregulated type III peroxidase genes, of which some are involved in lignin biogenesis, and jasmonic acid response genes, and phloroglucinol staining supported the activation of lignification by the LS treatment. As jasmonic acid-mediated lignification is a typical reaction to cell wall damage, it is possible that LS induces cell wall loosening, which can trigger cell wall damage response. Thus, LS is a unique chemical for modification of cell wall and morphology through changes in cell wall architecture.

Recent environmental issues have increased demand for bioresources as stable and sustainable industrial materials. Plant biomass is one of the most abundant land bioresources, the majority of which is accumulated as biopolymers, such as cellulose, hemicellulose and lignin, found in plant cell walls. Cellulose and hemicellulose polysaccharides have been identified as sugar resources for biofuels and other biomaterials^1^ and hence the plant cell wall is an important determinant not only for plant morphology through regulation of cell shape, but also for utilization of plant biomass through degradation to obtain sugar resources.

In order to increase total sugar yield from plant biomass, much effort has been made to improve both biosynthesis and decomposition of plant biomass^2^. Genetic modification has been widely tested as a method to alter biosynthesis of plant biomass^3^,^4^. One important target is lignin biosynthesis, because lignin content is generally negatively correlated with the degradation efficiency of plant biomass^5^. Recently the alteration of lignin content and/or its composition without negative effects on plant growth has been established, and it has been shown that such modification effectively increases enzymatic saccharification efficiency^6^. This result indicates that artificial design based on knowledge of gene function is a feasible strategy for modification of plant biomass. However, genetic manipulation requires adequate molecular information of target genes and established biotechnological methods, such as transformation technology, to modify target genes. These requirements make it difficult to apply genetic modification to non-model plants.

To overcome these problems, a promising solution is a chemical biological approach^7^,^8^. The screening of a chemical library is an effective way to identify novel interaction between chemicals and specific biological events and, in combination with genetics, chemical biology is now greatly contributing to understanding of plant molecular biological systems, including cell wall biosynthesis, the cytoskeleton, hormone biosynthesis and signaling, gravitropism, pathogenesis, and endomembrane trafficking^7^,^8^. Another attractive aspect of chemical biology is the possibility of exploiting the potential of identified chemicals for an application in question. Chemicals with high specificity for target molecules can be converted into useful tools to control particular biological events. All these aspects suggest that the chemical biological approach is a fruitful strategy with which to modify properties of plant cell walls without need for genetic modification and that in the future it can be applied to non-model industrial plants.

It has been reported that chemical screening to target plant cell morphology can be effective to isolate chemicals affecting plant cell wall biogenesis^9^,^10^,^11^. Here, we report the identification of novel small molecules with activity that changes the properties of plant cell walls. Through chemical screening using BY-2 suspension culture cells, we successfully identify lasalocid sodium (LS), also known as X537A, which is a carboxylic acid ionophore^12^,^13^, as a strong effector of change to the enzymatic saccharification efficiency of plant cell walls. This chemical probably affected the shape of the cell through regulation of cell wall loosening, which may be partly explained by up-regulation of peroxidase activity. Additionally, transcriptome analysis and cell wall analysis suggest that LS-induced abnormal cell wall loosening can trigger cell wall damage response in plants. Taken together, our results demonstrate great potential of the chemical biological approach to further cell wall engineering.

## Results

### Isolation of lasalocid sodium (LS) as a chemical affecting cell wall properties

**Figure 1:**
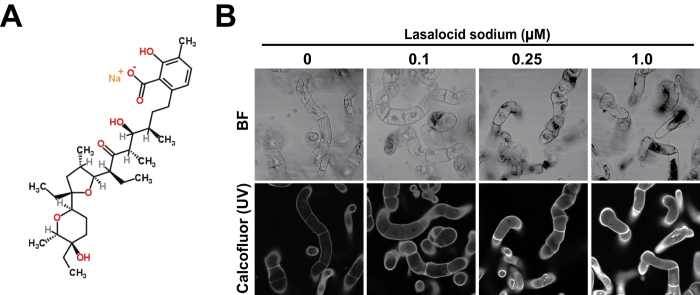
Dose-dependent effects of lasalocid sodium (LS) on morphology of tobacco BY-2 suspension cultured cells. (**A**) Molecular structure of LS. (**B**) Cell morphology of BY-2 cells after LS treatment. BY-2 cells were grown for 2 days in medium containing LS at the indicated concentrations, and then stained with calcofluor. Images were taken by confocal laser scanning microscope. BF, bright field. UV, ultra-violet.

To identify novel small molecules that affect properties of the plant cell wall, we screened the chemical library LATCA (Library of AcTive Compounds in Arabidopsis), which consists of 4,086 compounds that have bioactivity in *Arabidopsis* (http://cutlerlab.blogspot.jp/2008/05/latca.html). Chemical compounds from the LATCA library were added at a final concentration of 250 nM to a liquid culture of tobacco BY-2 suspension cells in 96-well plates one day after subculture. After a 2-day incubation, microscopic images of the chemical-treated BY-2 cells were obtained using a custom fluorescent microscope for a multiple well plate to select compounds that altered cell morphology (primary screening). As a result, we obtained 22 candidates (ex. Supplementary Fig. 1). We used calcofluor white, a specific dye that stains β-glucan that is present in cellulose, the main component of the cell wall, to observe the walls of BY-2 cells treated with candidate chemicals. This constituted the secondary screening (Supplementary Fig. 2A). Intensity profiles obtained from fluorescent images generated by confocal laser scanning microscopy revealed that 7 compounds strongly affected the calcofluor signal (Supplementary Fig. 2B), suggesting that these were candidates with potential to alter the cell wall. Of them, LS (Fig. 1A) showed activity that increased intensity of the calcofluor signal in a dose-dependent manner (Fig. 1B). LS is a carboxylic acid ionophore that binds divalent and monovalent cations, and is used as an antibacterial and an anti-coccidial agent^12^,^13^. However, how it affects plant cell walls is still unknown and therefore we carried out the following experiments to determine its action.

### LS changed cell wall thickness without obvious alteration to wall quantity and sugar composition

**Figure 2:**
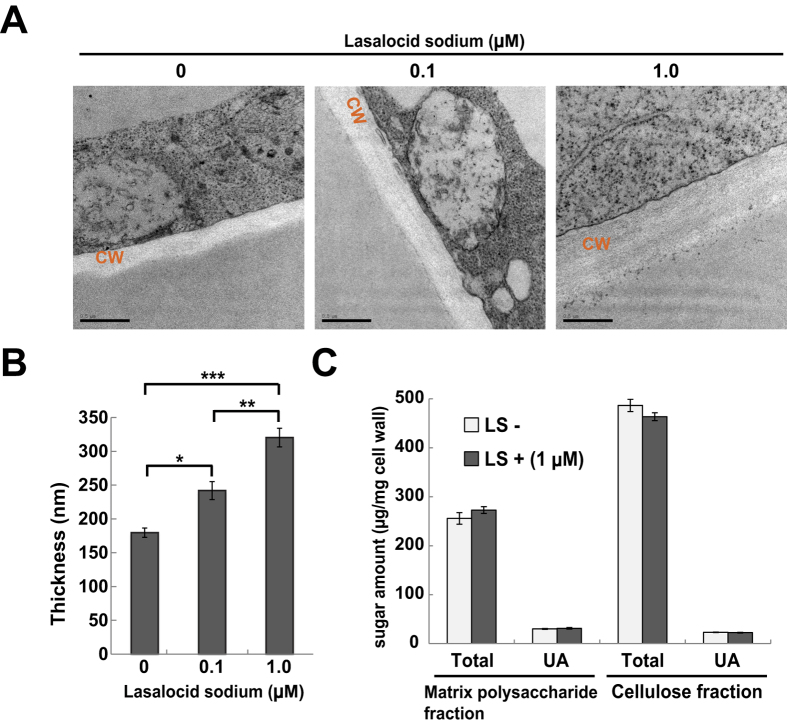
Effects of LS treatment on thickness and sugar content of cell wall. (**A**) Electron micrograph of cell walls of tobacco BY-2 cells treated with LS. Bar = 0.5 μm. CW, cell wall. (**B**) Cell wall thickness of tobacco BY-2 cells treated with LS. n = 100 per each. Asterisks indicate significant differences by Tukey-Kramer test (*p < 10^−3^, **p < 10^−4^ and ***p < 10^−11^). (**C**) Determination of sugar content in cell walls isolated from tobacco BY-2 cells treated with or without 1 μM LS. Total, total sugar. UA, uronic acid. LS, lasalocid sodium.

To investigate the effect of LS on cell wall structure, we observed BY-2 cell walls by transmission electron microscopy after LS treatment (Fig. 2A). Cell wall thickness increased in a dose-dependent manner, consistent with observations obtained from the confocal microscope. Thickness when treated with 1.0 μM LS was approximately twice that of non-treated cells (Fig. 2B). Solid-state NMR analysis showed that LS treatment hardly changed the cellulose crystallinity of the cell wall (Supplementary Fig. 3). This result indicated that change in wall thickness was not related to cellulose crystallinity.

Next, we checked the quantity of the cell wall and its cellulose, hemicellulose and pectin content with and without LS treatment. No significant differences were found in the amount of cell wall fraction or in the contents of each cell wall component between these samples (Fig. 2C and Supplementary Table 1). Analysis of the sugar composition of the matrix polysaccharide fraction also showed that the amounts of sugars in the LS treated samples were comparable to the non-treated samples (Table 1). Therefore, increase in cell wall thickness by LS does not result from changes in the amount or the chemical composition of the cell wall.

### LS increased saccharification efficiency

**Figure 3:**
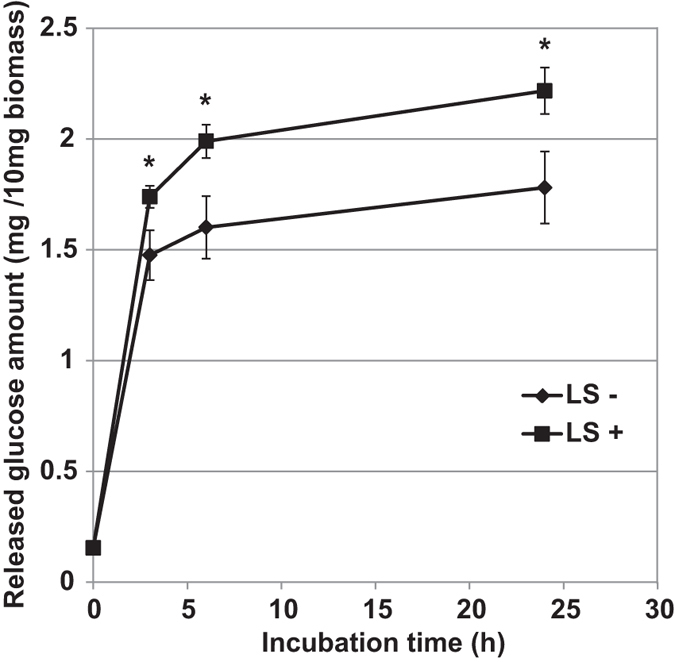
LS enhanced enzymatic saccharification efficiency of BY-2 cell walls. Isolated cell wall fractions from tobacco BY-2 cells treated with or without 1 μM LS were subjected to enzymatic saccharification analysis. Amount of glucose released during incubation in the enzyme solution containing cellulase and cellobiase is shown (n = 3). Bars indicate standard deviation. LS, lasalocid sodium. Asterisks indicate significant differences by Student’s t-test (p < 0.05).

To further analyze the effect of LS on properties of the cell wall, we investigated enzymatic saccharification efficiency of the cell wall material. Preparations from BY-2 cells with and without chemical treatment were subjected to enzymatic saccharification analysis. Results showed that LS-treated samples released approximately 1.2 times as much glucose as non-treated samples (Fig. 3). This increase was found from early stages of incubation with the enzymes. Taken together with observations that LS affected neither cellulose crystallinity nor compositional properties of the cell wall (Fig. 2C and Supplementary Fig. 3 and Table 1), the increase in enzymatic saccharification efficiency by the treatment is caused by changes in structural properties of cell wall components other than cellulose crystallinity.

### Effects of LS treatment on *Arabidopsis* seedlings

**Figure 4:**
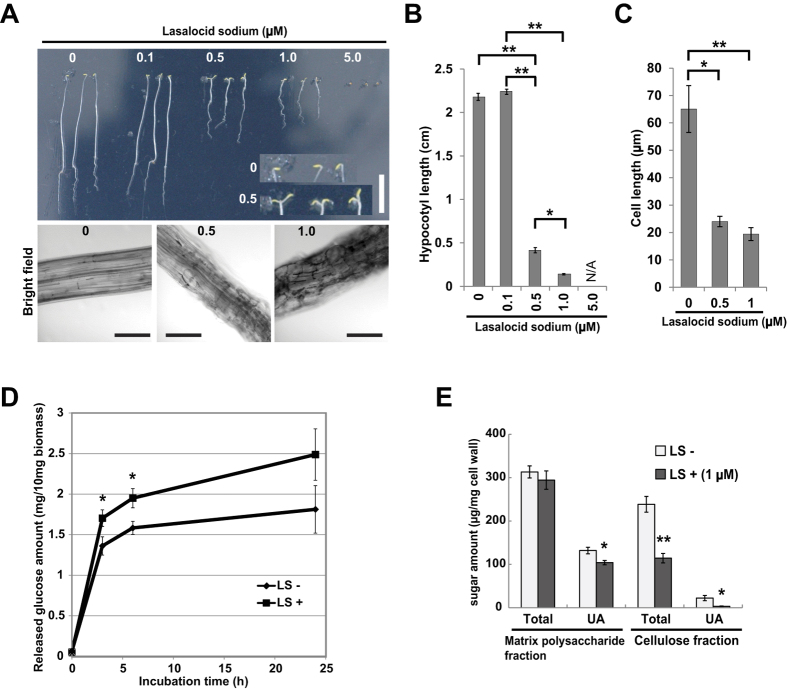
Effects of LS on growth of *Arabidopsis* seedlings. (**A**) Eight-day-old seedlings grown in medium containing LS at the indicated concentrations under dark conditions. Inset of upper panel shows close-up views of shoot apex regions of the seedlings grown in the presence of 0 and 0.5 μM LS. White bar = 1 cm. Black bars = 50 μm. (**B**) and (**C**) Hypocotyl (**B**) and cell (**C**) length of the seedlings shown in (**A**). Vertical cell lengths were measured. NA, not available. Asterisks indicate significant differences by Tukey-Kramer test (*p < 10^−7^ and **p < 10^−13^ in B, *p < 0.01 and **p < 0.001 in **C**). (**D**) LS also enhanced enzymatic saccharification efficiency of *Arabidopsis* seedlings. Isolated cell wall fractions from *Arabidopsis* seedlings treated with or without 1 μM LS were subjected to enzymatic saccharification analysis. Amount of glucose released during incubation in the enzyme solution containing cellulase and cellobiase is shown (n = 3). Bars indicate standard deviation. LS, lasalocid sodium. Asterisks indicate significant differences by Student’s t-test (p < 0.05). (**E**) Determination of sugar content in cell walls isolated from *Arabidopsis* seedlings treated with or without 1 μM LS. Total, total sugar. UA, uronic acid. LS, lasalocid sodium. Asterisks indicate significant differences by Student’s t-test (*p < 0.05 and **p < 0.01) between LS − and LS+.

To investigate effects of LS on plant growth, *Arabidopsis thaliana* seeds were germinated and grown continuously on MS agar medium containing 0, 0.1, 0.5, 1.0 or 5.0 μM of LS under dark conditions. Germination was severely inhibited by 5.0 μM, and elongation of hypocotyls was affected on medium containing 0.5 and 1.0 μM (upper panels in Fig. 4A,B). Abnormal-shaped cells and reduced cell length were observed in hypocotyls grown in the presence of 0.5 and 1.0 μM LS, (lower panels in Fig. 4A,C), suggesting that inhibition of hypocotyl elongation is mainly attributed to abnormal cell elongation and reduction of cell length. Moreover, we found that LS induced opening of cotyledons even in the dark, when cotyledons generally remain closed (Fig. 4A). We further checked enzymatic saccharification efficiency of *Arabidopsis* samples treated with or without LS. As with the BY-2 cells, chemical-treated samples showed higher amounts of released glucose compared with non-treated samples (approximately 1.3 times) from early stages of incubation with the enzymes (Fig. 4D). These results indicated that LS treatment can change cell wall properties not only in culture cells but also in plants.

After the result of increased enzymatic saccharification efficiency, we tested the possibility that LS can affect cell wall structures. Monosaccharide composition analysis on the cell wall matrix polysaccharide derived from LS-treated *Arabidopsis* seedlings revealed that LS treatment significantly increased the content of glucose, mainly derived from amorphous cellulose and hemicelluosic xyloglucan (Table 2, TFA-soluble fraction). Because xylose content was not affected by LS treatment (Table 2, TFA-soluble fraction), the glucose increase would be attributed to changes in amorphous cellulosic portion. In addition, in TFA-solubilized fraction of LS-treated samples, the uronic acids were decreased and the glucuronic acid levels were increased, suggesting altered pectic structures in LS-treated sample (Fig. 4E and Table 2, TFA-solubilized fraction). Glucuronic acids are known to be involved in rhamnogalacturonan II (RG-II) region of pectin^14^, thus we checked the effects of boron application in combination with LS on primary root length. Boron is an important element which crosslinks RG-II structures for dimerization to stabilize cell wall structures^14^, and excess amounts of boron would give a negative effect on root growth^15^. We measured primary root lengths of *Arabidopsis* plants grown on media containing boric acids, LS or both (Supplementary Fig. 4). The results showed that LS treatment did not affect the effects of boric acid application on root length (Supplementary Fig. 4), suggesting that boron-mediated dimerization of RG-II was not affected by LS treatment. In accordance with these observations, the ^11^B solid-state NMR spectra of whole BY-2 cells treated with or without LS exhibited identical symmetric peaks (Supplementary Fig. 5), indicating that LS did not influence the RG-II-borate tetrahedral complex. Taken together, LS treatment could affect properties of matrix polysaccharide, such as amorphous cellulose and pectic component other than RG-II structures.

### LS induced cell wall damage response in *Arabidopsis* plants

To examine the effects of LS on gene expression, we performed microarray analysis using 3-day-old dark-grown *Arabidopsis* seedlings treated with or without the chemical for 24 hours. The results showed that 1,430 and 1,526 transcripts were significantly increased and decreased, respectively, by LS treatment (threshold value of fold change is 1.5 and p < 0.05). Gene ontology analysis showed that up-regulated genes were enriched in extracellular, membrane-related and cell wall-related components in “Cellular Component” categories and that genes annotated with enzymatic activities and transporter activity were enriched in the “Molecular Component” categories (Supplementary Fig. 6). The most remarkable finding from these enrichment analyses is that, of the transcripts that were increased, 23 encode class III secretable peroxidase family proteins, a part of which are presumed to function in lignification^16^,^17^,^18^, and 27 encode jasmonic acid (JA)-related proteins including JA biosynthetic enzymes and JA-mediated signaling proteins (Supplementary Tables 2 and 3). Besides, some transcripts for lignin biosynthesis-related genes were also increased by LS treatment (Supplementary Table 4). Previous works have suggested that reactive oxygen species (ROS) production and JA signaling are important cross-talk modules to regulate cell wall damage-induced lignin biosynthesis^19^,^20^. Thus, it was possible that LS triggers cell wall damage response in plants.

**Figure 5:**
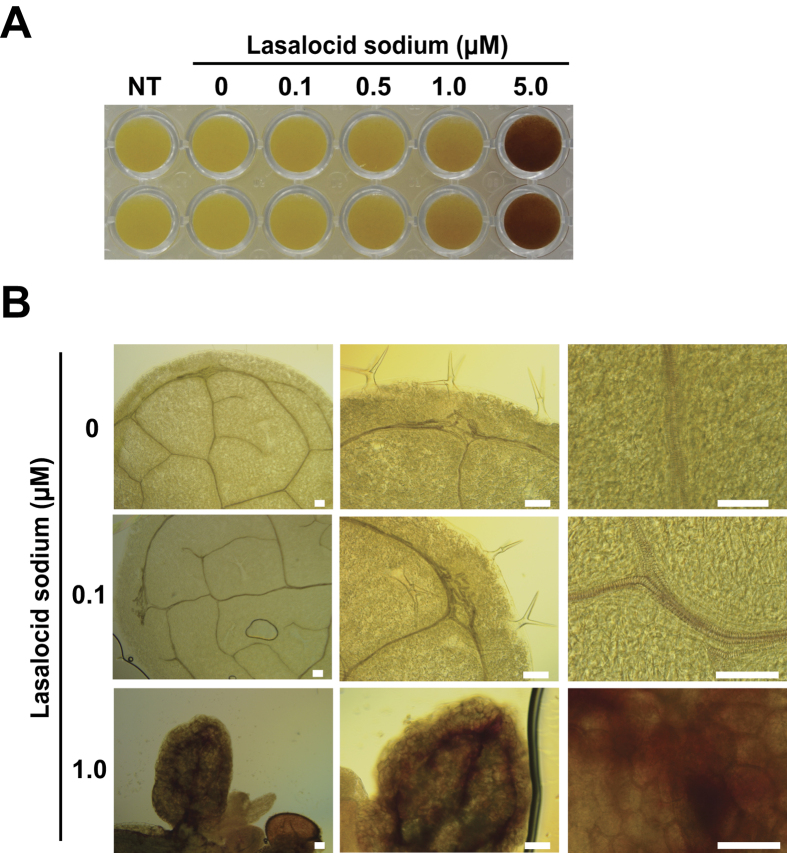
Increased peroxidase activity and lignin deposition caused by LS treatment. (**A**) DAB staining analysis of mm2d suspension cells treated with LS at the indicated concentrations. NT, no test. (**B**) Phloroglucinol staining of leaves in 14-day-old seedlings grown in medium containing LS at the indicated concentrations. Dark purple color indicates lignified tissues. Bars = 250 μm (**B**).

To check if high levels of peroxidase gene expression induced by LS are associated with increased peroxidase activity, mm2d *Arabidopsis* suspension cells were incubated with different concentrations of 0, 0.1, 0.5, 1.0 and 5.0 μM LS for 24 h, and then subjected to diaminobenzidine (DAB) staining analysis. The results showed that DAB staining signal increased as concentration of LS increased, indicating that the chemical activates peroxidase activity through up-regulation of peroxidase gene expression and releases ROS (Fig. 5A). Next, we checked whether LS treatment induced lignin accumulation by staining with phloroglucinol-HCl, which dyes lignin-containing tissues a red-violet color. We observed that phloroglucinol staining was more apparent in vascular tissues of plants grown on medium containing 1 μM LS compared with control plants (Fig. 5B). These facts show that LS increases peroxidase activity by up-regulation of class III peroxidase genes in plant cells, and that the increased peroxidase activity would increase lignification activity.

## Discussion

In this article, LS was isolated as a novel chemical that affected cell wall properties in plants. In BY-2 cells, LS treatment increased the thickness of the cell wall, but it had little effect on the sugar composition of the wall (Fig. 2, Supplementary Figs 3 and 4C, and Table 1), suggesting that LS induced the loosing of cell wall structure. LS-treated *Arabidopsis* showed changes in a part of cell wall architectural factors, e.g. increased amorphous cellulose and/or altered pectic components (Fig. 4E, Table 2), possibly related to cell wall expansion. Thus, it is supposed that in BY-2 cells calcofluor can enter through walls of cells treated with LS more readily than those of untreated ones, resulting in the higher calcofluor signals observed (Fig. 1). Similarly, high enzymatic saccharification efficiency in LS-treated BY-2 (Fig. 3) and *Arabidopsis* (Fig. 4) could be explained by increased accessibilities of enzymes after the loosing of cell walls.

LS induced abnormal cell morphology in *Arabidopsis* resulted in short hypocotyl length as well as early opening of cotyledons, when grown in the dark (Fig. 4). Cell morphology is determined by a balance between strength of the cell wall and turgor pressure, and looseness of the cell wall is an important regulator of cell elongation^21^. It is possible that LS disrupts this regulation, inducing abnormal cell morphology and inhibition of cell elongation. LS is a well-known ionophore and antibacterial agent^12^,^13^. One hypothesis is that LS also acts as an ionophore in plant cells and disturbs the concentration gradient of cations, e.g. calcium ions, between membranes. Disturbance of this gradient can trigger cellular responses associated with gene expression alteration^22^,^23^,^24^. The difference of effects on the wall sugar composition between LS-treated BY-2 cells and *Arabidopsis* (Fig. 4E, Tables 1 and 2) suggested that the first common response to LS at a cellular level is the loosing of cell wall structures, without alteration of cell wall monosaccharide composition. The alteration of matrix polysaccharides in *Arabidopsis* (Table 2) could occur as a result of the subsequent response at a tissue level. Calcium ions are known to mediate dimerization of homopolygalacturonan chains that are important for strength of the pectin^25^,^26^, thus this would account for the change in plant morphology together with the alteration in cell wall properties.

The microarray analysis revealed that class III peroxidase and JA-related genes are up-regulated by LS, leading to increased peroxidase activity (Fig. 5A and Supplementary Table 2). It is known that peroxidases have regulatory roles in plant cell elongation through regulation of the accumulation of reactive oxygen species (ROS) and the release of radicals^27^,^28^,^29^, thus treatment of LS can influence ROS production in plant cells (Fig. 5A). Recently, increases of ROS and/or JA production have been widely reported in *Arabidopsis* plants with cell wall damages^19^,^20^,^30^,^31^. Raggi *et al*.^31^ showed that growth defects in *qua2-1* mutant, in which a putative pectin methyltransferase is defective and levels of deesterified homogalacturonan were reduced^32^, can be partially explained with ROS accumulation by overexpression of *AtPRX71* encoding class III peroxidase^31^. Thus, based on the discussion above, we speculate that LS treatment would trigger cell wall damage response including ROS and JA production, resulting in lignin accumulation in plants (Fig. 5). Denness *et al*.^19^ showed that ROS production by cell wall damage requires calcium-dependent processes, because inhibitors of calcium signaling inhibited both ROS production and cell wall damage-induced lignin deposition^19^. It is possible that LS-induced imbalance of calcium ions between plasma membrane can be one of the direct initiators of early cell wall damage response, such as ROS and JA production. In addition, accumulation of ROS by upregulated peroxidases can inhibit cell expansion, as reported previously^27^,^28^,^29^, possible leading to abnormal cell morphology and inhibition of seedling growth (Figs 1 and 4).

Taken together, it is supposed that LS is a unique chemical to alter plant cell morphology through changes in cell wall architecture. Our data demonstrated that the changes in cell wall by LS treatment increased the enzymatic saccharification efficiency of plant cell wall materials (Figs 3 and 4C), suggesting an application potency of LS not only for the investigation of cell wall damage responses triggered by the imbalance of cations, but also for novel strategy of biomass usage. Further detailed analysis of the cell wall after LS treatment will clarify the molecular action of LS on the plant cell wall.

## Methods

### Plant materials and culture conditions

Tobacco BY-2 (*Nicotiana tabacum* L. cv. Bright Yellow 2) and *Arabidopsis* mm2d suspension culture cells were subcultured weekly with a 95-fold dilution and 45-fold dilution, respectively, in modified Linsmaier and Skoog medium as previously described^33^. The BY-2 and mm2d cell suspensions were agitated on a rotary shaker at 130 rpm at 27 °C and 22 °C, respectively, in the dark.

Seedlings of *Arabidopsis thaliana* ecotype Col-0 (Fig. 4A–C) were aseptically grown at 22 °C under dark conditions for 8 days on agar medium containing Murashige and Skoog (MS) salt mixture (Wako Pure Chemical Industries, Ltd, Osaka, Japan), Gamborg’s vitamin mixture (Sigma Chemical Co., St Lois, MO, US) and 0.8% (w/v) agar.

### Chemical screening

The LATCA chemical library comprised of 4086 compounds was provided by Dr. Sean Cutler (UC Riverside, USA). At 2 days after subculture, 60 μl of BY-2 cells were dispensed into 96-well plates and mixed with each chemical, pre-dissolved in dimethyl sulfoxide (DMSO), at a final concentration of 250 nM. The plates were incubated at 27 °C in the dark for 2 days. For observation, plates were placed onto the inverted platform of a fluorescence microscope (IX81, Olympus Co. Ltd, Tokyo, Japan) equipped with a cooled CCD camera head system (DP70, Olympus) (Supplementary Fig. 1). Images of the wells were semi-automatically acquired using LuminaVision software (Mitani-corp., Tokyo, Japan).

### LS treatment and cell wall staining of BY-2 cells

LS was dissolved in dimethyl sulfoxide to make a stock solution. It was added to the BY-2 cell culture one day after subculture at 0, 0.1, 0.25 or 1.0 μM final concentration. After a 48 h culture with the LS, BY-2 cells were stained with 0.001% (w/v) calcofluor white (BD, New Jersey, USA) for 20 min. After incubation, the samples were observed using a LSM700 confocal laser scanning microscope (Zeiss, Oberkochen, Germany).

### Electron Microscopy

For transmission electron microscopy, the samples were fixed in 2% (v/v) glutaraldehyde, which was buffered with 20 mM sodium cacodylate at pH 7.0, for 20 h at 4 °C, and washed with the same buffer for 2 h at 4 °C. Then they were post-fixed with 2% (v/v) osmium tetroxide in 20 mM cacodylate buffer for 2 h at 4 °C. The fixed samples were run through an alcohol series and embedded in Spurr’s resin. Ultra-thin sections (70 nm thick) were cut with a diamond knife on an ULTRACUT E ultra-microtome (Leica, Wien, Austria), and transferred to Formvar-coated grids. They were double-stained with 1% (v/v) uranyl acetate for 20 min and with lead citrate solution for 15 min. After washing with distilled water, the samples were observed using a JEM-1200 EX transmission electron microscope (Jeol, Tokyo, Japan).

### Biomass characterization by solid-state NMR

BY-2 cells treated with LS were washed with distilled water three times, and then lyophilized. Lyophilized cells were ground to a powder with an Automill machine (Tokken, Inc., Chiba, Japan). This powder was washed with methanol at 323 K for 10 min three times. The pellet was then washed with a CHCl_3_/MeOH mixture (1:1 = v/v) at ambient temperature for 30 min followed by three washes in acetone, and then dried. The dry powder was resuspended in 1 mL of 50 mM acetate buffer (pH = 5.2) containing 1.5 wt% of sodium dodecyl sulfate and 5 mM of metabisulfate, shaken at ambient temperature for 1 day, and then centrifuged at 20,400 g for 5 min, before the supernatant was discarded. Pellets were washed in the buffer three times and resuspended in the same buffer containing 20 units of α-amylase (from Bacillus species). The mixtures were incubated while shaking for 24 h at 343 K, and finally the supernatant was discarded. Pellets were washed first with the buffer three times followed by three washes in distilled water, and then lyophilized. Lyophilized powders underwent the following solid-state NMR analysis. All solid-state NMR spectra were recorded with an Avance-III HD 500 spectrometer (Bruker, Billerica, USA), equipped with a double-resonance 4-mm magic-angle spinning (MAS) probe essentially similar to previous reports^34^,^35^,^36^. The chemical shifts were referenced to the carbonyl group of α-glycine (δ^13^C: 176.46 ppm). Cross-polarization magic-angle spinning (CP-MAS) was measured with a relaxation delay of 5 s, and contact times of 2.0 ms under a 12 kHz MAS frequency. For ^11^B solid-state NMR analysis, BY-2 cells treated with or without LS were washed with distilled water, and then lyophilized. ^11^B solid-state NMR spectra were recorded using whole lyophilized cells including cell wall material, proteins, and lipid. ^11^B solid-state NMR spectra were recorded by the Hahn-echo method with a recycling delay of 2 s and a MAS frequency of 10 kHz. The chemical shifts were referenced to saturated boric acid solution (δ^11^B: 0 ppm).

### Cell wall preparation, sugar content determination and glycosyl composition analysis

Cell wall material preparation, the determination of carbohydrate content in matrix polysaccharides and cellulose, and glycosyl composition analysis were performed by the method described previously^37^. Briefly, approximately 5 g of BY-2 cells and *Arabidopsis* seedlings treated with or without LS for 48 h were collected and frozen in liquid nitrogen. The samples were sonicated in 0.5 M potassium-phosphate buffer (pH7.0). After washing, the cell wall material was extracted first with chloroform/methanol (1:1, v/v), and then with phenol/acetic acid/water (2:1:1, v/v/v). The starch was hydrolyzed with α-amylase, and finally the cell wall materials were freeze-dried. To obtain the matrix polysaccharide fraction, the cell wall material was hydrolyzed with 2 M TFA. The TFA-insoluble material was hydrolyzed with H_2_SO_4_ to yield the cellulose fraction. The amount of total sugar and uronic acid in each fraction was determined by the phenol–sulfuric acid method and the *m*-hydroxybiphenyl assay respectively. The glycosyl composition of the matrix polysaccharide fraction was examined by gas–liquid chromatography; the obtained fraction was converted to the trimethylsilyl ether of the corresponding methylglycoside and analyzed on a DB-1 column (0.25 mm × 30 m: J & W Scientific Co., Folsom, CA, USA). For the detailed experimental conditions, please see Goué *et al*.^37^.

### Enzymatic saccharification analysis

For *Arabidopsis* seedling, the 5-day-old seedlings were soaked into a liquid medium (MS salt mixture and 1% (w/v) sucrose, pH 5.8) with or without 1 μM LS, and then incubated at 22 °C in the dark for 2 days. After the incubation, the samples were harvested and frozen in liquid nitrogen immediately. The BY-2 cell wall materials prepared as described above and *Arabidopsis* seedlings treated with LS were ground using an Automill machine TK-AM7 (Tokken Inc.) after freeze-drying, and then washed with water three times, followed by three washes with methanol. After hydrolysis of the starch by treatment with *Aspergillus oryzae* α-amylase (SIGMA, 62-11) at 25 °C for 48 h, the samples were incubated in an enzyme solution containing 0.1 mg/ml of 5:1 mixture of cellulase from *Trichoderma reesei* ATCC 26921 (SIGAM, C2730) and cellobiase from *Aspergillus niger* (SIGMA, C6105) at 45 °C. The samples were collected at 0, 3, 6, 24 h of the incubation, and the glucose concentration was determined using ‘Glucose CII-Test’ (a kit with glucose oxidase, Wako).

### Transcriptome profiling analysis

Seeds of *Arabidopsis thaliana* ecotype Col-0 were sterilized with sodium hypochlorite solution, and incubated in water in the dark at 4 °C for 4 days. Subsequently, the seeds were sown onto germination medium comprised of MS salt mixture, Gamborg’s vitamin mixture, 1% (w/v) sucrose and 0.8% (w/v) agar (pH 5.8) followed by incubation at 22 °C in the dark for 3 days. The 3-day-old seedlings were put into a liquid medium (MS salt mixture and 1% (w/v) sucrose, pH 5.8) with or without 1 μM LS, and then incubated at 22 °C in the dark for 24 h. After the incubation, the samples were harvested and frozen in liquid nitrogen immediately. Total RNA was extracted from them using Plant RNeasy mini kits (Qiagen). Methods of labeling, hybridization on Arabidopsis V4 microarrays (Agilent) and data analysis have been described previously^38^. One microgram of the total RNA was used for the labeling reaction. Significant differences were defined by *Welch*’s t-test (P < 0.05). Array data have been submitted to the GEO database at NCBI as GSE79562.

### DAB (3,3′-Diaminobenzidine) staining

LS of each concentration was added to 1.5 ml mm2d cell culture at 3 days after subculture. After 24 h culture, cells were collected in a 1.5 ml tube, put on a bench for a few minutes and then the supernatant was removed. For staining, three drops of Simplestain DAB solution (Nichirei Biosciences) were added to the cells and mixed well by tapping.

### Phloroglucinol staining

*Arabidopsis* plants were grown for 14 days on germination medium containing 0, 0.1, and 1.0 μM LS under 16 h day/8 h night, and then dehydrated and decolorized by several changes of ethanol. Saturated phloroglucinol solution in 20% HCl was applied to the bleached plants. Stained images were obtained using an upright microscope.

### Measurement of root growth

*Arabidopsis* plants were grown for 5 days on germination medium containing half MS salt mixture, 1% sucrose and 0.8% (w/v) agar and transferred onto each growth condition under 16 h day/8 h night. Five days later, root lengths were measured.

## Additional Information

**How to cite this article**: Okubo-Kurihara, E. *et al*. Modification of plant cell wall structure accompanied by enhancement of saccharification efficiency using a chemical, lasalocid sodium. *Sci. Rep.* **6**, 34602; doi: 10.1038/srep34602 (2016).

## Supplementary Material

Supplementary Information

## Figures and Tables

**Table 1:** Glycosyl composition of matrix polysaccharide in BY-2 cell wall materials.

	Sugar amount (mol%)			
	TFA-0 μM L.S.	TFA-1 μM L.S.	H2SO4-0 μM L.S.	H2SO4-1 μM L.S.
Arabinose	28.5 ± 7.8	24.8 ± 3.6	0.0	0.0
Rhamnose	5.6 ± 1.4	6.1 ± 1.1	0.0	0.0
Fucose	0.0	0.0	0.0	0.0
Xylose	15.2 ± 1.3	17.3 ± 1.6	0.0	0.0
Mannose	6.1 ± 0.9	6.3 ± 0.4	4.5 ± 0.1	4.0 ± 0.5
Galactose	22.4 ± 4.1	22.4 ± 2.6	0.5 ± 0.1	0.3 ± 0.2
Glucose	13.0 ± 3.2	14.6 ± 1.8	93.1 ± 0.3	93.0 ± 0.4
Galacturonic acid	8.2 ± 1.6	9.8 ± 1.6	1.9 ± 0.4	2.6 ± 0.2
Glucuronic acid	1.0 ± 0.7	1.6 ± 0.2	0.0	0.0

Three technical replicates were performed. L.S., Lasalocid Sodium. TFA, TFA-soluble fraction; H_2_SO_4_, Hydrolyzed TFA-insoluble fraction with H_2_SO_4_.

**Table 2:** Glycosyl composition of matrix polysaccharide in *Arabidopsis* cell wall materials.

	Sugar amount (mol%)			
	TFA-0 μM L.S.	TFA-1 μM L.S.	H2SO4-0 μM L.S.	H2SO4-1 μM L.S.
Arabinose	16.4 ± 1.6	19.3 ± 1.5	0.0	0.0
Rhamnose	8.9 ± 0.7	7.6 ± 0.7	0.0	0.0
Fucose	2.0 ± 0.3	1.6 ± 0.2	0.0	0.0
Xylose	9.7 ± 0.7	9.6 ± 1.0	0.0	0.0
Mannose	1.3 ± 0.1	1.4 ± 0.2	3.1 ± 0.4	2.8 ± 0.3
Galactose	19.2 ± 1.1	16.6 ± 0.6	0.0	0.0
Glucose	8.2 ± 0.9	12.7 ± 0.5*	96.6 ± 0.4	97.2 ± 0.3
Galacturonic acid	33.1 ± 5.3	28.5 ± 2.4	0.3 ± 0.3	0.0
Glucuronic acid	1.1 ± 0.1	2.8 ± 0.3*	0.0	0.0

Three technical replicates were performed. L.S., Lasalocid Sodium. TFA, TFA-soluble fraction; H_2_SO_4_, Hydrolyzed TFA-insoluble fraction with H_2_SO_4_.

^*^Significantly different between 0 μM and 1 μM LS treatment (Student t-test, p < 0.05).
