# Supplementary material for: Modification of plant cell wall structure accompanied by enhancement of saccharification efficiency using a chemical, lasalocid sodium
Source: Sci Rep. 2016 Oct 3;6:34602. doi: 10.1038/srep34602 (PMC5046155; doi:10.1038/srep34602)
Supplement: Supplementary Information [file srep34602-s1.pdf]

Supplementary Information

Modification of plant cell wall structure accompanied by enhancement of saccharification efficiency using a chemical, lasalocid sodium.

Emiko Okubo-Kurihara, Misato Ohtani, Yukio Kurihara, Koichi Kakegawa, Megumi Kobayashi, Noriko Nagata, Takanori Komatsu, Jun Kikuchi, Sean Cutler, Taku Demura, Minami Matsui

Supplementary Table 1.

Amonts of cell wall fraction (dry weight of cell wall fraction (mg)/fresh cell weight (g))

	1	2	3	Mean	SD	t-test p
0 mM LA	2.17	1.97	1.96	2.0333333	0.1184624	0.1626752
1 mM LA	2.06	2.26	2.36	2.2266667	0.1527525	

Supplementary Table 2.

Upregulated peroxidase genes by lasalocid sodium treatment.

AGI_typeIII	ratio (L.S.+/L.S.-)	p-value
AT1G30870	2.390825532	0.045500028
AT1G77100	6.638529875	0.002147679
AT1G44970	2.333377774	0.002444302
AT2G18980	2.426538478	0.006670234
AT2G18150	2.121583451	0.026212585
AT2G38390	1.812834284	0.041681493
AT2G38380	3.849472037	0.001373
AT2G43480	2.104803024	0.011065619
AT2G35380	2.417221501	0.015028364
AT2G39040	3.394290759	0.023237499
AT3G21770	1.899422178	0.003658363
AT3G32980	2.133679202	0.019458402
AT3G28200	2.331834848	0.002198158
AT3G49120	2.212956789	0.010617783
AT3G01190	1.977342613	0.048400546
AT4G11290	2.260416256	0.037882252
AT4G16270	3.989415454	0.004370708
AT4G08770	2.635296974	0.032251225
AT5G19890	3.477466309	0.029312709
AT5G15180	3.418082304	9.93782E-05
AT5G42180	1.90501404	0.012719948
AT5G06720	3.156687091	0.003947764
AT5G22410	2.248570067	0.042149973

Supplementary Table 3.

Upregulated jasmonic acid related genes by lasalocid sodium treatment.

AGI	ratio (L.S.+/L.S.-)	p-value	description
AT1G17420	2.256731804	0.0171956	lipoxygenase 3
AT1G18570	1.580353634	0.0232525	myb domain protein 51
AT1G32640	1.565434482	2.04E-05	Basic helix-loop-helix (bHLH) DNA-binding family protein
AT2G06050	1.524838284	0.0366132	oxophytodienoate-reductase 3
AT2G18950	1.795980687	0.0426036	homogentisate phytyltransferase 1
AT2G25000	2.48145509	0.0018395	WRKY DNA-binding protein 60
AT2G27690	3.625572602	0.0001889	cytochrome P450
AT2G30040	1.536868868	0.030085	mitogen-activated protein kinase kinase kinase 14
AT2G30770	2.085796685	0.0078628	cytochrome P450
AT2G31180	3.023821266	0.0245391	myb domain protein 14
AT2G34810	2.250651546	0.0402208	FAD-binding Berberine family protein
AT3G25770	3.766125522	0.0481833	allene oxide cyclase 2
AT3G25780	3.282504691	0.0270983	allene oxide cyclase 3
AT3G51450	1.564467099	0.0005903	Calcium-dependent phosphotriesterase superfamily protein
AT4G10390	2.967398587	0.0305883	Protein kinase superfamily protein
AT4G22880	2.06999455	0.0422655	leucoanthocyanidin dioxygenase
AT4G23600	4.600489294	0.0014856	Tyrosine transaminase family protein
AT4G23810	1.839693721	0.0380228	WRKY family transcription factor
AT4G31500	2.283363731	0.0233859	cytochrome P450
AT5G24780	1.547538126	0.0486586	vegetative storage protein 1
AT5G26170	2.737356241	0.0012468	WRKY DNA-binding protein 50
AT5G38710	1.508122501	0.0100583	Methylenetetrahydrofolate reductase family protein
AT5G46050	3.018716187	0.0056722	peptide transporter 3
AT5G47120	1.504890195	0.0417658	BAX inhibitor 1
AT5G53750	2.384315054	0.0404669	CBS domain-containing protein
AT5G58670	1.722406645	0.0273352	phospholipase C1
AT5G61420	8.38997306	0.0194284	myb domain protein 28

Supplementary Table 4.

Upregulated lignin biosynthetic genes by lasalocid sodium treatment.

*Gene ID	Ratio(L+/L-)	p-value	Description
AT1G51680	1.551724713	0.003499	4-coumarate:CoA ligase
AT2G40890	1.574141	0.019855	Cytochrome P450
AT4G34050	1.50418588	0.001167	Caffeoyl coenzyme A O-methyltransferase
AT4G36220	1.478068039	0.016125	Ferulic acid 5-hydroxylase
AT5G54160	1.544012507	0.010573	Caffeic acid/5-hydroxyferulic acid O-methyltransferase
AT5G42180	1.90501404	0.01272	Lignin peroxidase
AT5G66690	2.508664193	0.002748	Monolignol glucosyltransferase
AT5G26310	2.377828162	0.001325	Monolignol glucosyltransferase

*These genes are selected from Zhao et al. *Plant Cell* **25**, 3976-3987 (2013).

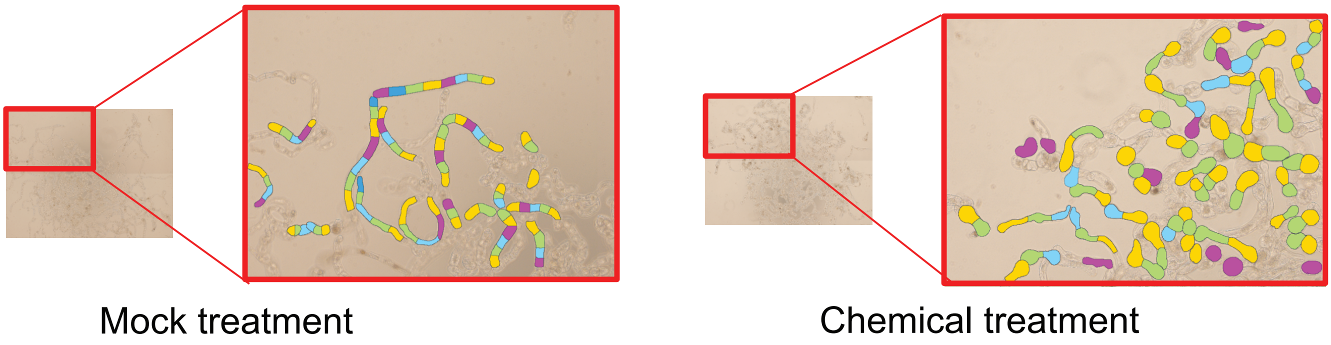

Supplementary Figure 1. Typical cell shape in the control tobacco BY-2 cells (left) and an example of abnormal cell shapes observed after chemical treatment (right). In the panel, cells were colored to make the shapes clear.

A

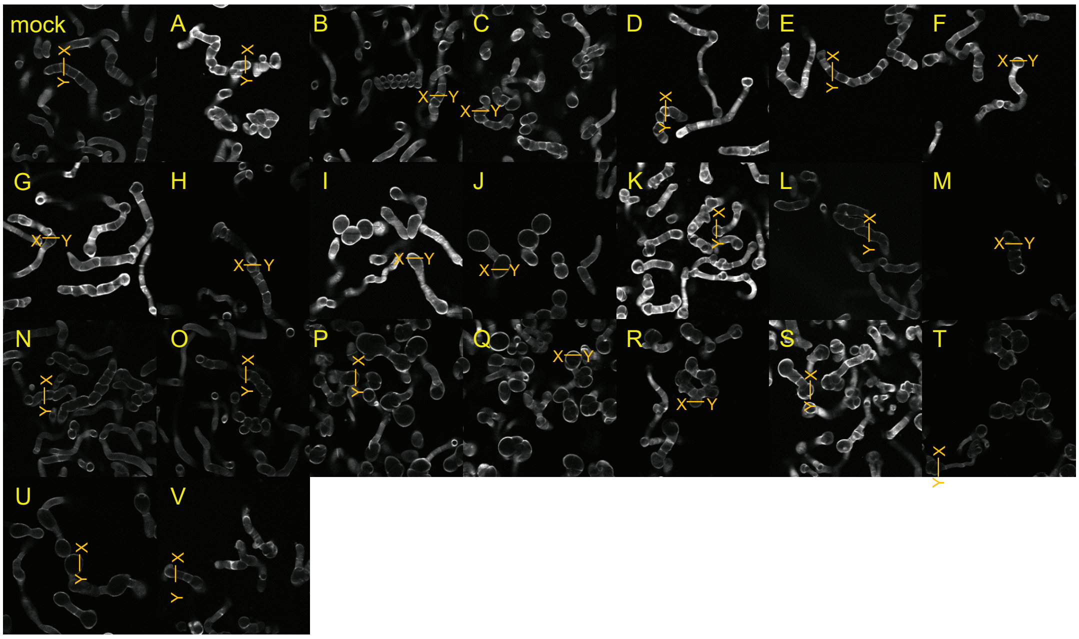

B

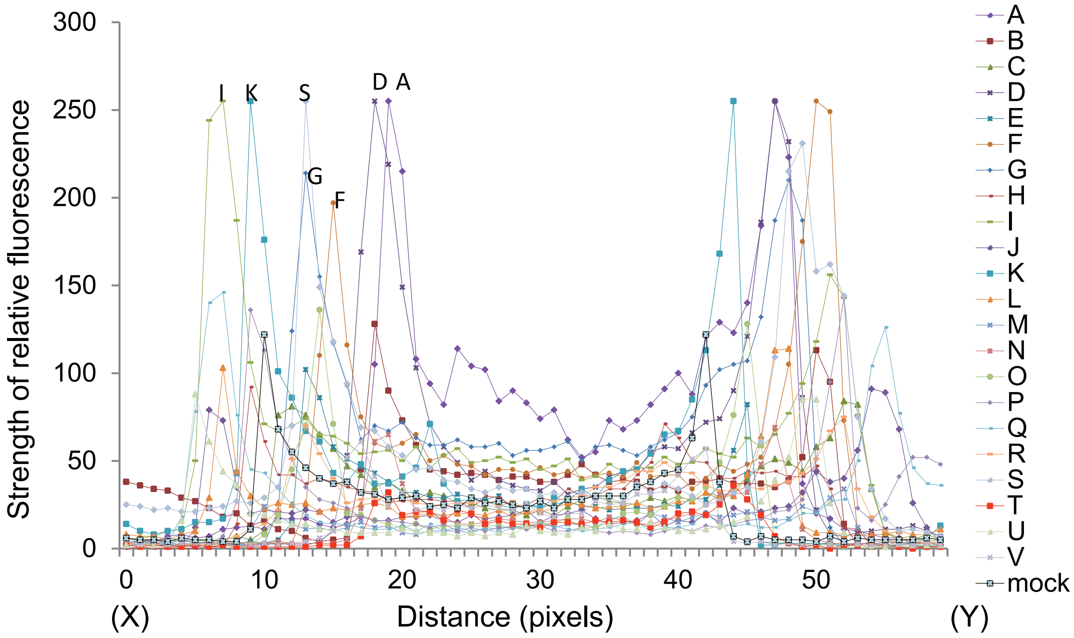

Supplementary Figure 2. Second screening of chemicals based on calcofluor staining.
(A) Confocal microscope images of BY-2 cells treated with 22 candidate compounds A-V after calcofluor staining.
(B) Calcofluor signal intensities of X-Y incisions in (A).

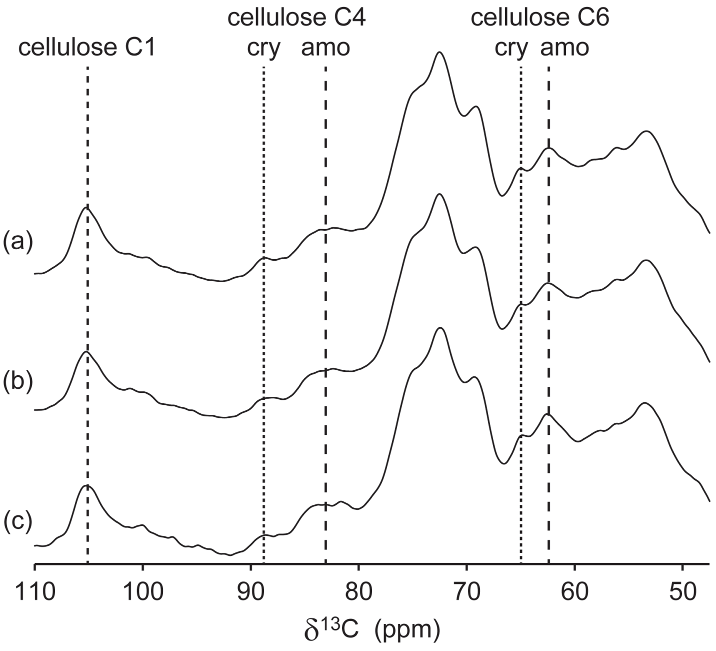

Supplementary Figure 3. CP-MAS spectra of cell walls of BY-2 cells treated with or without lasalocid sodium. (a) The cell walls of BY-2 cells treated with 1 μM lasalocid sodium. (b) The cell walls of BY-2 cells treated with 0.1 μM lasalocid sodium. (c) Control. Signals indicated with dotted lines were annotated with cellulose according to previous reports (Mori et al., 2012 and Komatsu et al., 2013). cry: Iβ-crystalline cellulose, amo: amorphous cellulose.

Komatsu, T. & Kikuchi, J. Selective Signal Detection in Solid-State NMR Using Rotor-Synchronized Dipolar Dephasing for the Analysis of Hemicellulose in Lignocellulosic Biomass. *J. Phys. Chem. Lett.* 4, 2279-2283 (2013).

Mori, T., Chikayama, E., Tsuboi, Y., Ishida, N., Shisa, N., Noritake, Y., Moriya, S. & Kikuchi, J. Exploring the conformational space of amorphous cellulose using NMR chemical shifts. *Carbohyd. Polym.* 90, 1197-1203 (2012).

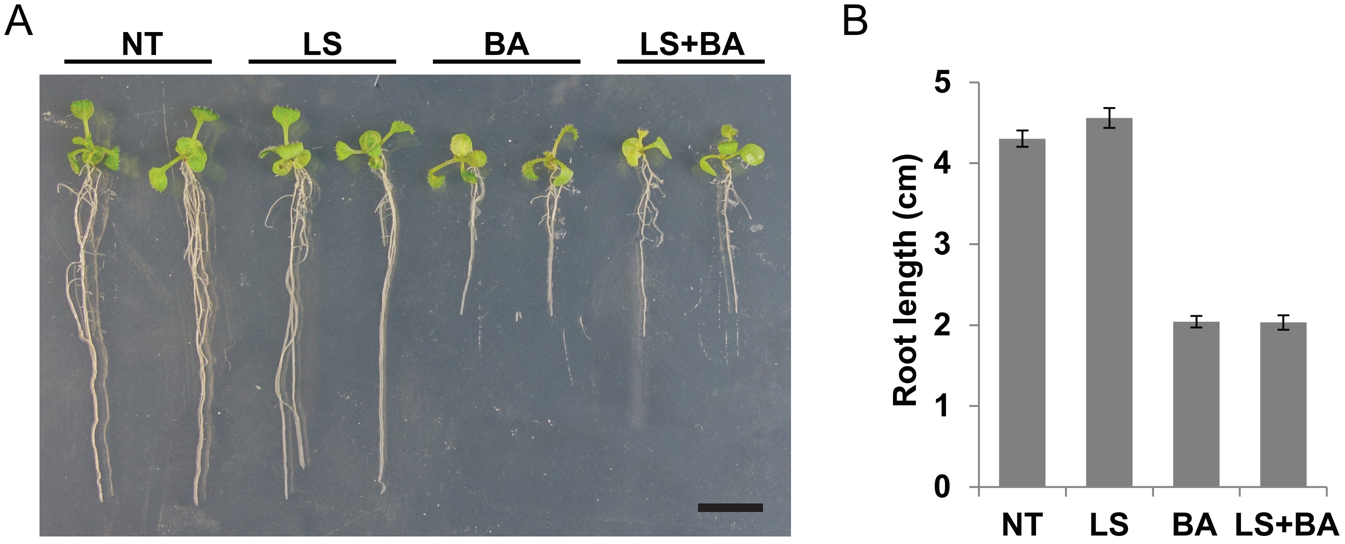

Supplementary Figure 4. Root length mesurement in Arabidopsis seedlings grown on medium containing boron and/or LS.
(A) Five-day-old Arabidopsis seedlings were transferred onto four kinds of growth conditions; Non-Treated (NT), 0.5 μM Lasalocid Sodium (LS), 5 mM Boric Acids (BA) and 0.5 μM Lasalocid Sodium and 5 mM Boric Acids (LS+B), and the root lengths were mesured 5 days later. Scale bar = 1 cm.
(B) Root length. n = 30.

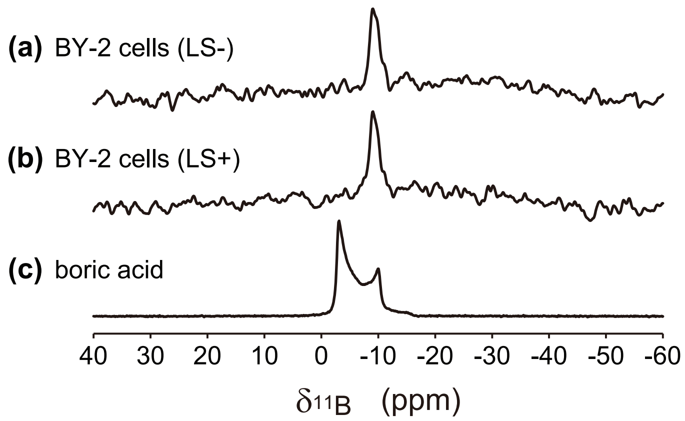

Supplementary Figure 5. 11B MAS NMR spectra of BY-2 cells to characterize insoluble boron in the cell wall. (a) 11B MAS NMR spectra of BY-2 cells without any treatment. (b) 11B MAS NMR spectra of BY-2 cells treated with X mM lasalocid sodium. There were identical symmetric peaks at -9.0 ppm in (a) and (b). These symmetric line shapes indicated that boron existed in tetrahedral configuration, because the local electric field gradient became small compared with other configurations such as for boric acid (c) [ref]. These symmetric 11B peaks were considered to be dimeric rhamnogalacturonan II (RG-II) -borate complex, in which two monomeric RG-IIs are cross-linked by borate–diol esterification. These spectra indicate there is no significant difference in structure of RG-II.

[ref] Kameda, T., Ishii, T., Matsunaga, T., and Ashida, J., 11B Solid-state NMR Investigation of the Rhamnogalacturonan II-borate Complex in Plant Cell Walls, Analytical Science (2006) 22, 321-323 (doi.org/10.2116/analsci.22.321).

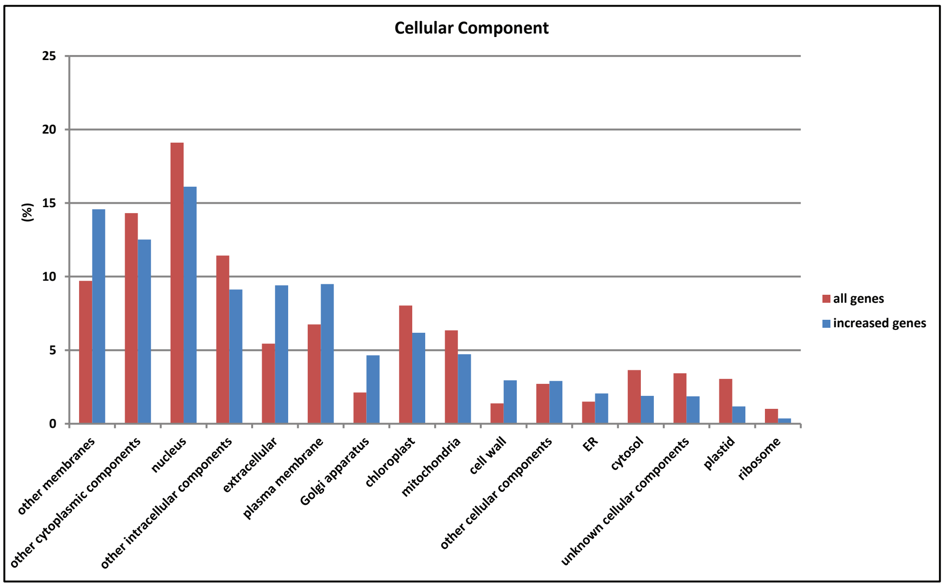

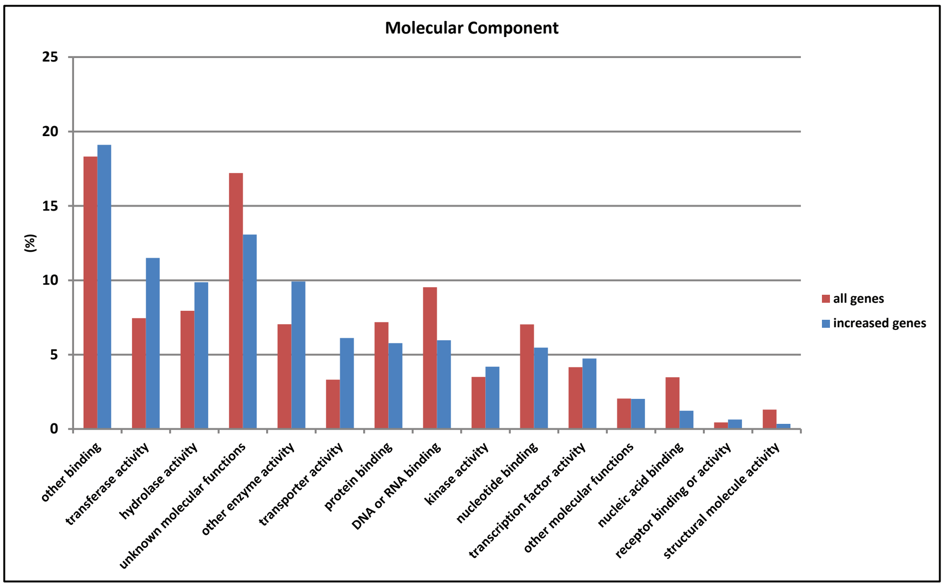

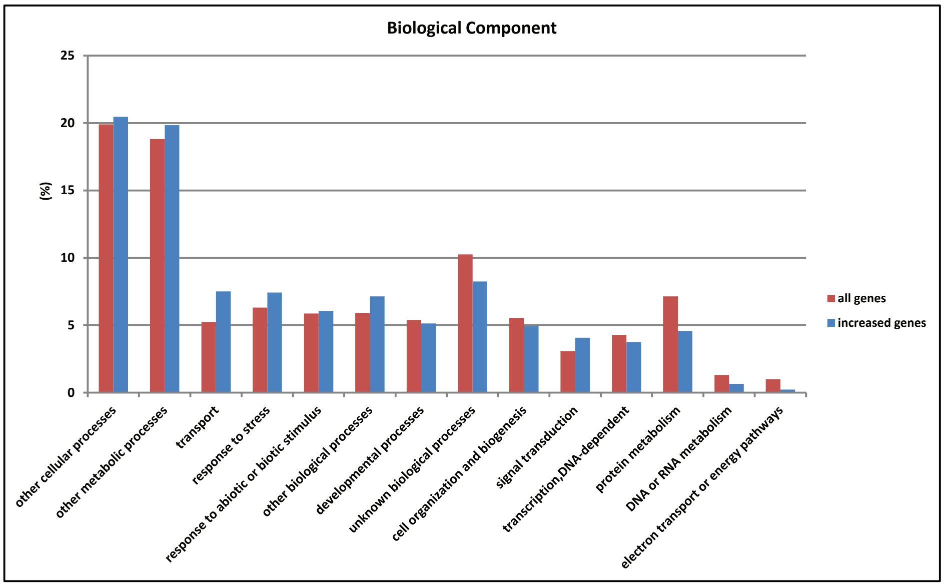

Supplementry Figure 6. Gene ontology analysis of genes with increased expression in presence of lasalocid sodium in microarray. The genes were compared with all genes with annotations in the Arabidopsis genome.
